# A Slight Adjustment of the Nutri-Score Nutrient Profiling System Could Help to Better Reflect the European Dietary Guidelines Regarding Nuts

**DOI:** 10.3390/nu14132668

**Published:** 2022-06-27

**Authors:** Véronique Braesco, Emilio Ros, Azmina Govindji, Clélia Bianchi, Lise Becqueriaux, Belinda Quick

**Affiliations:** 1 VAB-Nutrition, 63100 Clermont-Ferrand, France; clelia.bianchi@vab-nutrition.com; 2 Lipid Clinic, Endocrinology and Nutrition Service, Institut d’Investigacions Biomediques August Pi Sunyer, Hospital Clinic, University of Barcelona, 08036 Barcelona, Spain; eros@clinic.cat; 3 CIBER Fisiopatología de la Obesidad y Nutrición (CIBEROBN), Instituto de Salud Carlos III, 28029 Madrid, Spain; 4 Azmina Nutrition, London, UK; azmina@azminanutrition.com; 5 General Mills, Bell Institute of Health and Nutrition, Minneapolis, MN 55427, USA; lise.becqueriaux@genmills.com (L.B.); belinda.quick@genmills.com (B.Q.)

**Keywords:** Front-of-Pack labelling, nutri-score, nuts, nutrient profiling systems, dietary guidelines, intakes

## Abstract

The consumption of nuts remains low among European populations despite widespread inclusion as a recommended food group across European dietary guidelines. Front-of-Pack nutrition labelling systems are designed to support consumers make healthier choices and to stimulate product improvement, thus representing a pivotal opportunity to reduce the gap between intakes and recommendations. This study examined how the Nutri-Score algorithm treats nuts and nut-containing products and tested whether slight adjustments could better recognise and motivate nut inclusion in foods and diets. The nutritional score (ScN) and corresponding Nutri-Score letter of 68 nuts and nut-containing products were calculated, using the initial algorithm and slight adjustments, where nut weight was doubled (S1), saturated fats (S2) or energy (S3) from nuts were discounted, or saturated fats were replaced by the saturated fats/lipid ratio (S4). The correlation between the nuts’ content and the ScN was moderate for the initial algorithm (R^2^ = 0.34) and S1 (R^2^ = 0.36), but improved for S2, S3 and S4 (R^2^ = 0.54, 0.55 and 0.52, respectively). Four plain nuts, initially labelled as “B” or “C” obtained a Nutri-Score “A” with S2, S3 and S4. Slight adjustments could better align the Nutri-Score with food-based dietary guidelines, reassure consumers on healthfulness of nuts and nut-containing products, whilst incentivising the inclusion of nuts in diverse foods.

## 1. Introduction

Nuts encompass tree nuts (almonds, Brazil nuts, cashews, hazelnuts, macadamias, pecans, pine nuts, pistachios, and walnuts) and peanuts, which botanically are legumes but have a nutrient profile similar to that of tree nuts. Nuts have a beneficial nutrient profile, which underlies the recognised health-promoting properties of their frequent consumption [1]. They are the natural plant food that is second richest in fat after vegetable oils (Table 1). The fatty acids of nuts, however, are mostly unsaturated (monounsaturated (MUFA) and polyunsaturated (PUFA)), hence their saturated fatty acid (SFA) content is low (range, 4 to 16%). Nuts also contain other reputedly salutary macronutrients and phytochemicals. They are a sizable source of vegetable protein (between 8 and 25% of energy). They are also a good source of dietary fibre, ranging from 5.7 to 12.5 g per 100 g, and also contain micronutrients with recognised health benefits, such as the B-vitamin folate and tocopherols (vitamin E). Among nut phytochemicals, two molecules stand out because of their abundance and biological activity: polyphenols, which are powerful antioxidants, and plant sterols, natural inhibitors of intestinal cholesterol absorption. Like most plant foods, plain nuts are practically devoid of sodium, but are rich sources of beneficial minerals, such as calcium, magnesium, and potassium [1].

Many studies have examined the consumption of nuts in relation to cardiovascular outcomes. A recent meta-analysis of 19 prospective cohort studies [2] found an inverse association between total nut consumption and cardiovascular diseases (CVD) with mean risk reductions ranging from 15 to 24% for CVD incidence, CVD mortality, coronary heart disease (CHD) incidence, CHD mortality, and stroke mortality, when comparing highest vs. lowest consumption categories. A recent report of three large prospective studies described that decreasing nut consumption increased the risk of CVD and stroke [3]. Additionally of note, low nut and seed consumption was a critical dietary factor contributing to overall CVD mortality in large population surveys in the U.S. [4], Europe [5] and Latin America [6]. Finally, a landmark nutrition intervention trial, the PREDIMED study, had an arm of Mediterranean diet supplemented with mixed nuts (30 g/day of a mixture of walnuts, almonds and hazelnuts) and the results after intervention for nearly 5 years showed a 28% reduction in incident CVD, including a 45% reduction in stroke risk, in comparison with a control low-fat diet [7]. Thus, consistent data from numerous large prospective studies and meta-analyses plus striking evidence from a major randomised controlled trial (RCT) suggest that nuts are powerful cardioprotective foods. These effects can be ascribed to the myriad of beneficial nutrients contained in nuts, which also contribute to heart health by improving CVD risk factors.

Evidence from prospective studies also suggests that nut consumption is associated with a reduction of adverse cardiometabolic outcomes, such as the incidence of hypertension, obesity and, to a lesser extent, Type 2 diabetes [8]. Of note, concerning diabetes, only walnuts among tree nuts have been associated with a lower risk [1]. Of particular interest is the fact that regularly eating such high energy-dense foods as nuts does not promote a positive energy balance, but is associated with less weight gain, diminished body fat, and a lower incidence of overweightness/obesity with time, as shown in a recent meta-analysis of prospective studies, while an absence of undue weight gain has been consistently observed in nut feeding trials [9].

Numerous well-conducted RCTs have examined the effect of nut diets on blood lipids and lipoproteins, as well as blood pressure (BP). The most comprehensive meta-analysis computed data from 61 trials, and the summary results were that nut consumption (per serving/day) significantly reduced total cholesterol (−4.7 mg/dL), LDL-cholesterol (−4.8 mg/dL), and triglycerides (−2.2 mg/dL), but had no effect on HDL-cholesterol [10]. This same meta-analysis examined the effects of nut diets on BP and found no effect, although BP changes were usually secondary outcomes of small-sized RCTs. However, a recent large RCT of a walnut-enriched diet in older individuals for outcomes of changes in 24-h ambulatory BP (the gold standard of BP measurements) reported a mean 8.5 mm Hg reduction of systolic BP in hypertensive participants after intervention for 2 years [11].

Nut consumption also has been largely reported for its beneficial impact on several non-cardiovascular health outcomes. Although based exclusively on observational evidence, a recent meta-analysis of 51 studies suggests that frequent nut consumption may be modestly associated with a reduced total cancer incidence and mortality [12]. Data from 14 epidemiological studies and 8 RCTs assessing the effects of nut-enriched diets on cognitive function has recently been reviewed [13]; the authors recognise that in general the quality of the evidence was low, but studies targeting populations at higher risk of cognitive decline tended to have positive results [1].

Exposure to nuts in relation to all-cause mortality has been examined in many large cohort studies. A recent meta-analysis summarised data from 18 prospective studies and concluded on a 19% reduced risk for all-cause mortality for high, compared to low, nut consumption [14].

Nutrient density and the recognised health benefits of nuts make them a recommended food group in dietary guidelines worldwide, and especially in those issued in European countries by both national authorities (Supplemental Table S1) or by scientific and healthcare societies or associations (see example for the United Kingdom, Supplemental Table S2). A daily intake is generally recommended, usually between 15 and 30 g, which roughly corresponds to a “handful” per day. This range encompasses the optimal level of intake of 21 g/day that has been defined as the appropriate level of exposure for minimising risk from all-causes death in the Global Burden of Disease Study [15].

Yet, nut consumption in the general population is far from this optimal level. In Europe, mean intakes in adults were estimated to range between 3 (Eastern Europe) to 5 g/day (Western Europe) [15]. Using food consumption data from national nutritional surveys, Mertens et al. showed that only 5% of Danish, 7% of Czech, 1% of Italian and 3% of French consumed at least 15 g/day of nuts and seeds. In each of these four countries, less than 25% of adults reported consuming nuts and seeds [16]. Similar trends were observed in Germany, where mean nut and seed intakes were 5 g/day in men and 4 g/day in women, with less than 50% of adults reporting any nut consumption [17].

This sizable disparity between nut recommendations and actual consumption is a public health issue and corresponding actions should be undertaken to bridge this gap [18]. In such a strategy, three key levers might be combined: (1) promoting the daily consumption of more plain nuts, (2) increasing the nut content of nut-containing products already on the market, and (3) enabling the development of new nut-containing products.

Front-of-Pack Nutrition Labelling (FOPNL) is of particular importance in relation to this purpose. This policy tool has two main objectives: to guide consumers towards healthier dietary choices and to incentivise food industries to develop and improve products with healthier nutrient profiles [19,20]. FOPNL is defined as “*nutrition information in the principal field of vision on food and drinks packaging. It could express the overall nutritional value of a food, by using some or all of the information from the nutrition declaration and/or other nutritional elements, to be applied on all products or only on products complying with certain nutritional criteria*” [19]. FOPNL can be either informative by highlighting the content in nutrients or ingredients, or interpretative, when based on a nutrient profiling system (NPS), which allows for the ranking and categorisation of foods according to their nutritional quality, evaluated using predefined criteria [21]. FOPNL has advanced in the last few years in Europe and a harmonised mandatory system is currently being defined by the European Commission (EC) [22]. The Nutri-Score, a graded and colour-coded indicator, has been largely studied, especially for its potential to guide consumers towards healthier choices [23,24], and is one of the interpretative FOPNL under consideration by the EC [22]. If it had to be endorsed in several European countries, the Nutri-Score should be aligned with national food-based dietary guidelines in Europe, notably those related to nuts, before being further extended. The Nutri-Score system is currently recommended in seven countries officially engaged in the Nutri-Score: France, Belgium, Luxembourg, Switzerland, Germany, The Netherlands, and Spain [25].

The aim of this study was to examine how Nutri-Score values nuts and nut-containing products and to elaborate and test scenarios consisting of slight adjustments of the Nutri-Score nutrient profiling system that could better rate plain nuts and the nut content of food products. These adjustments were made with the objective of suitably reflecting the beneficial contributions of nuts and nut-containing products while keeping the fundamentals of the Nutri-Score.

**Table 1:** Average nutrient composition of tree nuts and peanuts (per 100 g).

Nut Type	Energy (KJ)	Protein (g)	Fibre (g)	Fat (g)	SFA (g)	MUFA (g)	PUFA (g)	LA (g)	ALA (g)	Plant Sterols (mg)	Poly Phenols (mg)	Folate (µg)	Calcium (mg)	Magnesium (mg)	Sodium (mg)	Potassium (mg)
Almond	2469	22.6	12.5	51.3	4.1	31.5	12.2	12.2	0.00	162	287	44	269	270	2.6	733
Brazil nut	2874	16.9	6.4	66.1	16	24.5	24.4	20.5	0.05	72	244	22	160	376	3	659
Cashew	2610	20.5	5.7	49	8.9	27.3	7.8	7.7	0.15	120	233	69	45	260	8	565
Hazelnut	2548	17	11.6	56.9	4.8	45.7	7.9	7.8	0.09	115	671	113	114	163	2.6	680
Macadamia	3096	9.3	8.6	75.8	11.8	58.9	1.4	1.3	0.21	119	126	10	70	118	5.2	363
Peanut	2536	26.1	8.6	49.1	8.4	24.4	15.6	15.6	0.00	126	406	240	92	168	8.8	705
Pecan	3012	11.3	8.3	72.6	6.6	40.8	21.6	20.6	1.00	113	1284	22	70	121	1	410
Pine nut	2905	16.2	10	65	5.5	18.8	34.1	33.2	0.16	120	58	34	16	251	9	597
Pistachio	2460	21.7	10.1	47.4	5.5	25.0	14.0	13.2	0.25	272	1420	49	104	106	6	977
Walnut	2912	15.7	6.7	67.3	6.5	8.9	47.2	38.1	9.08	143	1579	98	98	158	2.6	441

Abbreviations: SFA, saturated fatty acids; MUFA, monounsaturated fatty acids; PUFA, polyunsaturated fatty acids; LA, linoleic acid; ALA, α-linolenic acid. Sources: Energy, protein, fibre, fat, SFA and sodium from ANSES, 2020 [26]; MUFA, PUFA, LA, ALA, and Plant sterols from Kornsteiner-Krenn et al., 2013 [27]; Polyphenols from Neveu et al., 2010 [28]; Folate, Calcium, Magnesium and Potassium from USDA, 20.19 [29].

## 2. Materials and Methods

### 2.1. The Nutri-Score

The Nutri-Score is a FOPNL system that has been endorsed by the French authorities since 2017 [30]. The Nutri-Score label is a graphic scale composed of 5 classes (expressed by a colour and a letter from dark green “A” (higher nutritional quality) to dark orange “E” (lower nutritional quality) [31,32]. Nutri-Score labelling is not mandatory in Europe, but has been recommended by governmental authorities in France, Belgium, Luxembourg, Switzerland, Germany, The Netherlands, and Spain [25]. In January 2021, these seven countries, officially engaged in the Nutri-Score, have established various committees aimed at providing independent advice on potential evidence-based updates for the current Nutri-Score algorithm, taking into account scientific knowledge, public health issues and being in synergy with food based dietary guidelines [25]. The European Commission, as part of its Farm to Fork strategy [22], announced that it will propose harmonised, mandatory FOPNL by the end of 2022. This initiative was opened for consultation in December 2021 and included the Nutri-Score amongst the proposals outlined. Responsibility for calculation of the Nutri-Score rests with food manufacturers, who must be registered, and follow the guidance and conditions of use published by authorities [32].

**Figure 1:**
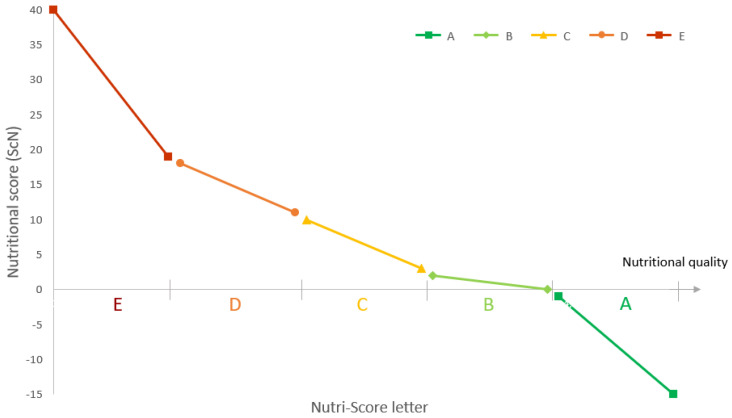
The decrease of the nutritional score (ScN) of the Nutri-Score NPS corresponds to an increase of the nutritional quality, and thus to a better Nutri-Score letter.

The Nutri-Score algorithm is based on the UK Food Standards Agency NPS [33], with modifications of the initial algorithm for the specific cases of cheese, added fats, and beverages. The reference base for the nutritional score calculation is 100 g or 100 mL [31,32]. The Nutri-Score NPS is a combination of negative and positive points. Negative points are attributed to four selected ‘unfavourable’ elements: energy, sugars, saturated fatty acids (SFA), and sodium. Each of these four elements are scored from 0 to 10 points, making the score of unfavourable elements range from 0 to 40. Positive points are attributed to three selected ‘favourable’ elements: one grouping fruits, vegetables, pulses, nuts, and rapeseed, olive, and walnut oils (FVPNO); another grouping proteins; a third comprising fibres. Each of the three elements are scored 0 to 5 points, making the favourable score range from 0 to 15, except that, if the unfavourable points are above 11 and the FVPNO score is less than 5, protein points are not counted in the score, and thus the favourable score ranges from 0 to 10. Then, each product is given a nutritional score (ScN), calculated from “unfavourable points minus favourable points”, resulting in a numerical value ranging theoretically from −15 (higher nutritional quality) to +40 (lower nutritional quality). Ultimately, a coloured letter (from A to E, from the higher to the lower nutritional quality), is assigned according to the ScN and can be displayed as the FOPNL [31,32]. Figure 1 below illustrates that the higher the ScN, the further the letter in the series is, and the darker/redder the corresponding colour of the Nutri-Score letter is. Lowering the nutritional score (ScN) would therefore correspond to an improvement of the nutritional quality and potentially to an improved Nutri-Score letter. Moreover, the distribution of points within Nutri-Score letters is unequal. The methodology of the calculation of the ScN and the attribution of the corresponding letter for foods is provided in Supplemental Figure S1.

In the Nutri-Score NPS, the term “nuts” refers to walnut, hazelnut, pistachio, Brazil nut, cashew nut, pecan nut, peanut, almond, and chestnuts (corresponding to Eurocode group 7.20 and 7.40). Nuts are recognised by the positive element, together with fruits, vegetables, legumes, and rapeseed, olive, and walnut oils. Favourable points are given only when a food product contains >40% of one or more of these food categories combined (1 point for >40 to 60% of FVPNO, 2 points for >60 to 80% of FVPNO, and 5 points for >80% of FVPNO) [32].

### 2.2. Development of the Four Scenarios for Adjustment of the NPS of the Nutri-Score

Four scenarios (S1, S2, S3 and S4) have been constructed, taking the following elements into consideration:

- Retaining the fundamentals of the Nutri-Score,
  ○ only nutrients labelled in the nutritional declaration,
  ○ assessment per 100 g or 100 mL,
  ○ applicable across all product categories
- Precedent adaptations by the creators of the system e.g., the double counting of dry fruit
- Introduction of a single variation to the system, such that all other criteria, thresholds, and calculations remain identical to the original Nutri-Score
- Unlikely to affect the Nutri-Score of products that do not contain nuts.
- Practically achievable
- Has nutritional significance relative to the specificities of nuts
  This resulted in four variations, as follows:
  ○ Scenario 1 (S1) consists of multiplying the weight of nuts by 2 when calculating the amount of FVPNO in 100 g of food, as is currently done by the Nutri-Score algorithm for dried fruits, vegetables, and pulses [34]. This helps to better consider the nutritional density of dried fruits, vegetables, and pulses in spite of their higher energy density, so it seems logical to apply the same treatment to nuts. Thus, the calculation of nuts percentage in this scenario is the following:
    $$\%\mathrm{Nuts}\_S1=\left(\frac{\%\mathrm{nuts}\times 2}{\left(100-\%\mathrm{nuts}\right)+\left(\%\mathrm{nuts}\times 2\right))}\right)$$
    For each food, the %Nuts_S1 was then used to calculate the number of points in the FVPNO element of the nutritional score for S1. The corresponding ScN was identified as ScN_S1.
  ○ Scenario 2 (S2) consists of discounting the SFA content of nuts in the SFA component of the Nutri-Score NPS. Indeed, SFAs in nuts are accompanied by large amounts of MUFA and PUFA and should not be considered as isolated SFA from other sources, which do not bring nutritional benefits. For each food, the value used to calculate the number of points in the SFA element of the ScN_S2 corresponded to the SFA content from nuts subtracted from the total SFA content. To calculate the SFA content of nuts in each product, the SFA content of each nut was collected from the French food composition table CIQUAL [26], as depicted in Table 1. The SFA content from nuts in a food product containing *n* different nuts was calculated using the following formula
    $$\mathrm{SFA}\;\mathrm{from}\;\mathrm{nuts}=\overset{n}{\underset{i=1}{\sum }}\left(\%\;\mathrm{nut}\;i\times \mathrm{SFA}\;\mathrm{content}\;\mathrm{in}\;\mathrm{nut}\;i\right)$$
    The corresponding ScN was identified as ScN_S2.
  Scenario 3 (S3) consists of discounting the energy content of nuts in the energy density element of the Nutri-Score NPS. The energy in nuts comes largely from fat, and discounting it is an alternative way of considering the overall favourable quality of fat in nuts; furthermore, there is consistent evidence that, unlike the energy from most foods, the metabolisable energy from nuts is less than that determined by food chemistry measurements and predicted by Atwater factors [35,36,37,38]. To discount energy density from nuts in the energy density element of the Nutri-Score NPS, the same method was applied as the one described above for (S2) but replacing SFA with energy density. The corresponding ScN was identified as ScN_S3.
  Scenario (S4) consists of replacing the SFA component of the Nutri-Score NPS by the ratio SFA/lipids, which is used in the initial algorithm adaptation for added fats [32]. This acknowledges that the composition of nuts in terms of fatty acid content is not limited to SFA, which make up only a minor part of the total fatty acids of nuts. One point is attributed for an SFA/lipids ratio of 10% and the number of points increases by one each time the ratio increases by 6% until a maximum of 10 points (for a SFA/lipids ratio of 64% or more) [34]. The corresponding ScN was identified as ScN_S4.

### 2.3. Selection of Food Products to Test the Four Scenarios

A cross-category sampling of nuts and nut-containing products (nut content between 5 and 100%) was established in order to reflect the variability in nut content across nuts and nut-containing products on the market. This facilitated the assessment of products belonging to a variety of food categories, products with varying nut content (lower, intermediate, higher) and nuts and nut-containing products with various Nutri-Score letters (A to E). The term “nut content” relates to the weight percentage of nuts contained in a food product.

For nut-containing products, the data required to calculate the ScN (nutritional information and %FVPNO) were collected on major e-commerce websites from Europe in March 2021. For plain nuts, nutritional information was collected from the CIQUAL French Food composition tables (Table 1) [26].

A supplemental sample of products that did not contain nuts was built in order to assess for potential side impacts of each scenario on the ScN of a variety of commonly consumed products. The nutrient composition of these products was collected from the CIQUAL French Food composition table 2020 [26].

### 2.4. Calculations and Statistical Analyses

For each product, 5 ScNs were calculated. The first ScN corresponded to the initial NPS of the Nutri-Score without any modification. The four other ScNs corresponded to each scenario. For each ScN, the corresponding Nutri-Score letter was determined.

For each scenario, the number of products that decreased their ScN (corresponding to a better nutritional quality) and the number that improved their Nutri-Score letter were calculated. The mean change in the ScN was calculated for each scenario, overall and by food category. A reduced ScN does not necessarily equate to an upgraded Nutri-Score letter. Thus, the number of products that obtained an ScN corresponding to an improved nutritional quality in such a way that they were only 1 or 2 points away from an upgraded Nutri-Score letter (i.e., for which reformulation could potentially result in obtaining a better Nutri-Score letter) was evaluated. Simple linear regressions with nut content as the dependent variable and the change in ScN as the independent variable were performed for each scenario. Correlations (R^2^) were also calculated separately for products containing less or more than 40% nuts in weight. This 40% threshold was selected because the current Nutri-Score algorithm considers nuts in its FVPNO component only when above 40% in weight. The impact on other food products that did not contain nuts was evaluated by calculating the changes in ScN and the corresponding Nutri-Score letter following the application of each scenario.

All analyses were performed using Microsoft Excel 2019 and its complements (Macros Visual Basic and Analysis ToolPack, Microsoft, Redmond, WA, USA).

## 3. Results

### 3.1. Assessment of Nuts and Nut-Containing Products

A total of 68 food products containing from 5 to 100% nuts, as labelled in the ingredient list, were included in the assessment. These foods belonged to 15 different categories (in accordance with the CIQUAL French food composition table classification [26]). The four most represented food categories were “Cereal bars” (*n* = 16; 24%)), “Plain nuts” (*n* = 9; 13%), “Breakfast cereals” (*n* = 7; 10%), and “Salted nuts” (*n* = 7; 10%). The mean nut content was 47.2 ± 37.7%. For each food category, the number of products included in the database and the mean nuts content are provided in Table 2.

The supplemental list of products belonging to different food categories and that did not contain nuts was composed of eight food products: “Ham”, “Yoghurt”, “Avocado”, “Salmon”, “Bread”, “Butter”, “Olive oil”, and “Emmental”.

The main and the supplemental lists are both available in Supplemental Table S3.

### 3.2. Nuts and Nut-Containing Products in the Initial Nutri-Score Nutrient Profiling System

**Figure 2:**
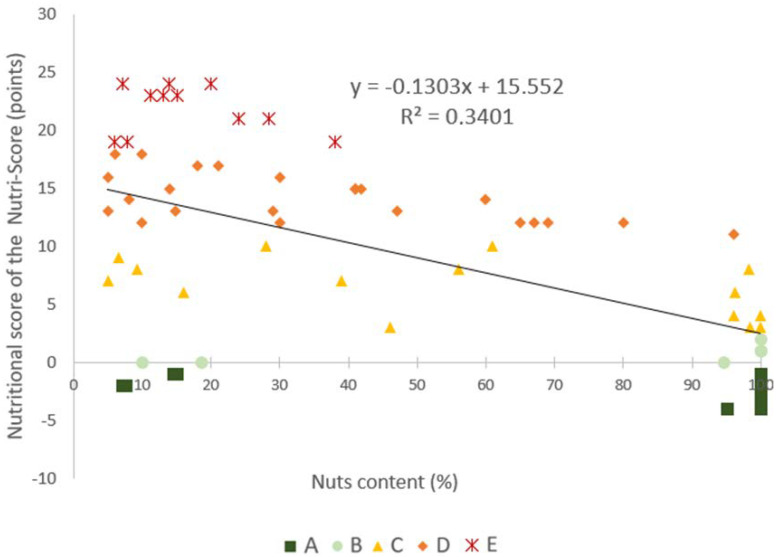
Correlation between the nuts content (%) and the corresponding nutritional score of the Nutri-Score (ScN) for 68 selected nuts and nut-containing products. Dark green squares correspond to products rated with a Nutri-Score letter “A”; Light green circles correspond to products rated with a Nutri-Score letter “B”; Yellow triangles to products rated with a Nutri-Score letter “C”; Light orange diamonds to those with a Nutri-Score letter “D”; and dark orange stars to those with a Nutri-Score letter “E”.

The mean ScN of the products included in the list was 9.4 ± 8.4 points, with all Nutri-Score letters represented. The correlation between the proportion of nuts (weight percentage) within each nut-containing food and their ScN was moderate (R^2^ = 0.34) (Figure 2), when calculated over the whole range of products. When the correlation was calculated separately for products with a nut proportion above and below 40% in weight, a much lower correlation was found for products containing less than 40% of nuts (R^2^ = 0.00036), compared to the correlation for products containing 40% nuts or more (R^2^ = 0.7247).

Regarding the nine plain nuts included in the list, five were given an ScN corresponding to the Nutri-Score letter “A” (almonds, hazelnuts, pecan nuts, pistachios, and walnuts), two received an ScN corresponding to the Nutri-Score letter “B” (cashews and peanuts) and two received the Nutri-Score letter “C” (Brazil nuts and macadamias). This means that not all plain nuts would be consistently labelled with the Nutri-Score letter “A”.

### 3.3. Application of the Four Scenarios to Nuts and Nut-Containing Products

**Figure 3:**
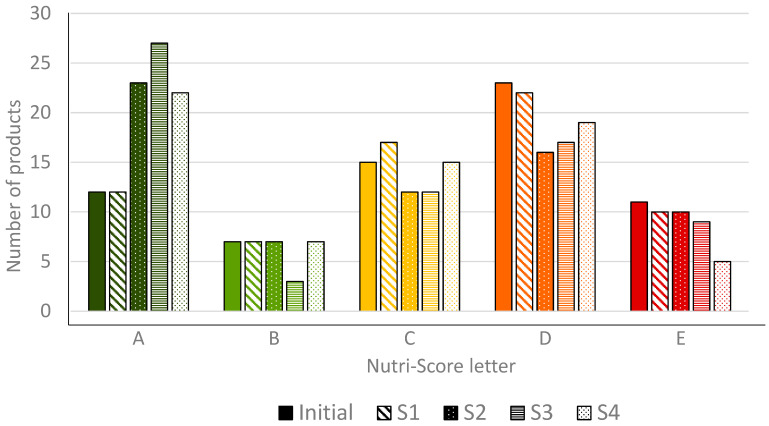
Distribution of a sample of 68 nuts and nut-containing food products in each Nutri-Score letter with the initial NPS of the Nutri-Score and with the application of S1, S2, S3 and S4.

The distribution of products across Nutri-Score letters with the initial Nutri-Score algorithm and with the application of S1, S2, S3 and S4 is presented in Figure 3. Detailed information of Nutri-Score letter and ScN of each food in each scenario is presented in Supplemental Table S3. Overall, S3 was the scenario that enabled more products to shift to an improved Nutri-Score letter. Regarding plain nuts, no change in their Nutri-Score letter was observed following the application of S1. However, following the application of S2, S3 and S4, all plain nuts presented an ScN that corresponded to the Nutri-Score letter “A”.

The number of products that obtained an ScN corresponding to a better nutritional quality (i.e., decrease in ScN of at least 1 point) was 11, 52, 62 and 60 following the application of S1, S2, S3 and S4, respectively (Table 3). The smallest decrease was observed following the application of S1 (mean: −0.4 points) while scores decreased more (−3.3. to −3.8 points) with the other scenarios. When considering the products that obtained an ScN corresponding to better nutritional quality following the application of each scenario, the mean changes were −2.3, −4.3, −4.1 and −3.9 points for S1, S2, S3 and S4, respectively.

Overall, S2, S3 and S4 resulted in more changes in the Nutri-Score letter than S1. For some products that obtained an ScN corresponding to a better nutritional quality, some did not reach a better letter. This corresponded to 8, 27, 35 and 29 products for S1, S2, S3 and S4, respectively. However, some of these products are only one or two points away from a better Nutri-Score letter that could be reached with an appropriate reformulation. The number of such products was 4 with S1, 5 with S2, 8 with S3, and 8 with S4.

Importantly, except for S4, no scenario led to a significant improvement of the products which were initially attributed with a “E” letter: products that were labelled “E” in initial system (including cookies, confectionary, chocolate-nut spread, etc.) remained E with S1, S2 and S3.

### 3.4. Association between the Nut Content and Nutritional Score of the Nutri-Score following the Application of Each Scenario

**Figure 4:**
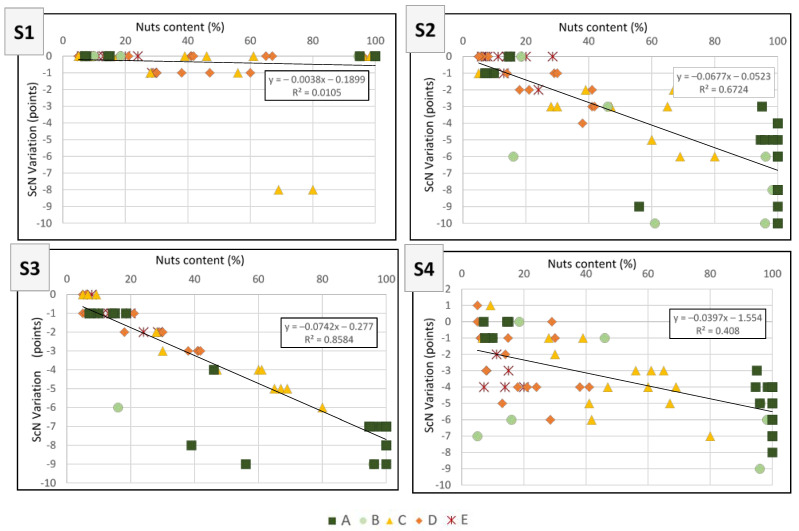
Association between nuts’ content as a percentage of weight and variation of the nutritional score of the Nutri-Score (ScN) for 68 selected nuts and nut-containing food products following the application of 4 modification scenarios to the Nutri-Score nutrient profiling system (where nut weight was doubled (**S1**), saturated fats (**S2**) or energy (**S3**) from nuts discounted, or saturated fats were replaced by the saturated fats/lipid ratio (**S4**)). Dark green squares correspond to products rated with a Nutri-Score letter “A”; Light green circles correspond to products rated with a Nutri-Score letter “B”; Yellow triangles to products rated with a Nutri-Score letter “C”; Light orange diamonds to those with a Nutri-Score letter “D”; and dark orange stars to those with a Nutri-Score letter “E”.

Figure 4 shows the linear regressions between the nut content of the 68 nuts and nut-containing products and the variation of the ScN following the application of S1, S2, S3 and S4. The correlations between nut content and variation in the ScN were high for S2 and S3 (R^2^ = 0.67 and 0.86 respectively), moderate for S4 (R^2^ = 0.41) and very low for S1 (R^2^ = 0.01). Finally, the correlation between nut content and the ScN was still moderate following the application of S1 (R^2^ = 0.36) when compared to the initial ScN (R^2^ = 0.34), but was higher following the application of the other three scenarios (R^2^ = 0.54, 0.55 and 0.52 for S2, S3 and S4, respectively) (Figure 4 and Table 4).

Correlations were also calculated separately for products with a nut proportion above or below 40% in weight, for the four scenarios. Data displayed in Table 5 suggest that the ScN value remains better correlated to the nut contents when the nut content is above vs below 40%, for all scenarios, as observed for the current Nutri-Score. When examining correlations between nut content and ScN variation, they are of similar magnitude whatever the nut content, for S2 and S3, while being overall stronger for S3 when compared to S2; this suggests that, for both these scenarios, the changes do not favour more the products with a proportion of nuts below vs. above 40%. For S4, these correlations were weak and were stronger for products with a higher proportion of nuts. For S1, these correlations are of little relevance, because very few products saw their score modified.

### 3.5. Assessment of the Impact of the Application of Each Scenario on the Nutri-Score Letter of Core Food Products That Did Not Contain Nuts

The application of S1, S2 and S3 did not modify the ScN and the Nutri-Score letter of food products that did not contain nuts, as modifications of the NPS only related to nuts for these three scenarios. However, the application of S4 resulted in three of the eight non-nut containing food products obtaining a worse Nutri-Score letter (ham, yoghurt and bread) (see Supplementary Table S3).

## 4. Discussion

Given the richness of nuts in beneficial nutrients, their positive influence on many health outcomes, and the evidence of low consumption levels in Europe, one would expect that the Nutri-Score FOPNL in use today would clearly appraise nuts and nut-containing foods such as to reflect their overall beneficial and nutritional qualities and to ensure consistency with food-based dietary guidelines. FOPNL could contribute to the three key levers to reduce the gap between nut intakes and recommendations, i.e., (1) promoting the daily consumption of more plain nuts, (2) increasing the nut content of nut-containing products already on the market, and (3) enabling the development of new nut-containing products. Although specific evidence is still lacking that nut intake would increase with nut-containing products being more appropriately scored, FOPNL, and particularly the Nutri-Score, have been shown to be able to favourably affect food purchases, with consumers more often selecting products that contribute to diet quality when a Nutri-Score letter is present [39,40].

Our results clearly show that, currently, plain nuts are not given the highest Nutri-Score letter and that there is only a moderate correlation between the nut content and the ScN in nut-containing products. Our modelling study shows that slight adjustments of the current Nutri-Score algorithm, such as discounting the SFA content or the energy density of nuts, results in a better correlation between the nut content and the ScN of the Nutri-Score, without improving the Nutri-Score letter of food products that were classified as “E” (i.e., worse nutritional quality). Interestingly, these modifications also resulted in attributing the best Nutri-Score letter to all plain nuts included in our database, which can help the consumer to identify all of them as healthier foods, and they did not affect the Nutri-Score of products that did not contain nuts. Finally, we showed that with viable and slight modifications, the improvements in the Nutri-Score of nuts and nut-containing food products can be better correlated to the nut content in foods, which was consistent with the objective of this study.

In the Nutri-Score NPS, nuts accrue N points unequivocally, in particular for their inherent energy density and SFA content, which obviously cannot be reformulated. Conversely, the accrual of P points does not account for most of the nutritional assets brought by nuts (MUFA, PUFA, B-vitamins, minerals, and polyphenols). This is further compounded for nut-containing products wherein the attribution of available protein points (from nuts, or other sources) is reliant on total product N points not exceeding 11 points [33]. This discrepancy in the Nutri-Score NPS is demonstrated by the fact that plain nuts such as, Brazil nuts, macadamias, peanuts, and pecans are rated with a Nutri-Score letter “B” or “C”.

Among the four scenarios of modifications of the Nutri-Score NPS tested in this work, not counting SFA (S2) or energy density (S3) from nuts in the calculation of the ScN of the Nutri-Score were the preferable options for a better consideration of nuts in the Nutri-Score NPS. Indeed, these modifications resulted in a high correlation between the nut content and the ScN, whilst not impacting products that do not contain nuts and keeping the fundamentals of the Nutri-Score NPS.

Both these adjustments would be nutritionally justified. Indeed, nuts are nutrient-dense foods, as their total fat content ranges from 44% (*w*/*w*) in cashews to 76% in macadamias. However, the fatty acids composition of nuts is of marked nutritional interest because their SFA content, which is penalising them, is low (4 to 16% of energy),while being associated with high amounts of unsaturated fatty acids: mostly MUFA in most nuts, similar amounts of MUFA and PUFA in Brazil nuts, and mostly PUFA in walnuts [1].

Notwithstanding the high energy density of nuts, there is consistent evidence that their consumption does not favour weight gain. Of particular interest is the fact that regularly eating nuts does not promote a positive energy balance, but is associated with less weight gain, diminished body fat, and a lower incidence of overweightness/obesity with time, as shown in a recent meta-analysis of prospective studies, while the absence of undue weight gain has been consistently observed in nut feeding trials [9]. In a meta-analysis of 55 clinical trials involving the daily consumption of nuts or nut-based snacks/meals (mean intake of 48.2 ± 20.8 g nuts/day) by 3811 adults for 3 weeks or more (mean duration of 13.8 ± 21.5 weeks), no changes in weight, BMI and waist circumference were observed [41]. A recent RCT showed that including peanuts as snacks in the habitual diet of Chinese adults at high risk of cardiometabolic diseases during 12 weeks in place of rice bars improved overall metabolic syndrome risk without promoting weight gain [42]. Major observational studies in Europe such as PREDIMED-Plus [43] or EPIC-PANACEA [44] have reached the same conclusion.

Several reasons for the absence of weight gain ensuing nut consumption have been considered. First, eating nuts increases satiety due to their high fat and fibre contents, with the suppression of hunger and promotion of fullness [1,9,45]. Second, nut ingestion entails impaired energy absorption efficiency in the gastrointestinal tract due to a reduced bioaccessibility of fat encased in nut cell walls, plus an increased binding of fatty acids in the gut by nut fibre, with attending increases in fecal fat losses [1,9]. This leads to an overestimation of the metabolisable energy of nuts by the widely used Atwater factors. Indeed, in various clinical trials, these factors overestimated the metabolisable energy of almonds [35], walnuts [36], and pistachios [37], and cashew nuts [38] by 32, 21, 5 and 16%, respectively. Finally, the high unsaturated fatty acid levels in nuts are believed to enhance fatty acid oxidation and increase thermogenesis and resting energy expenditure, which may also help mitigate weight gain [1,9,46]. Thus, the common concern that nut consumption contributes to weight gain and increased adiposity appears unwarranted. Such a neutral or even beneficial effect of nut consumption on adiposity is a fitting argument to discount the energy density of nuts in the Nutri-Score NPS.

Thus, there are relevant scientific arguments to discount SFA or energy density, as developed in scenario S2 or S3.

The recently established transnational governance including the seven countries officially engaged in the Nutri-Score has committed to consider “*evidence-based adjustments to the nutrient profiling system of Nutri-Score, taking into account scientific knowledge and public health issues in the nutritional field, in synergy with the food-based dietary guidelines* [47]. In their annual report, experts from the transnational governance identified a diet that is low in nuts and seeds to be of concern in Europe [48]. Our results suggest that both adjustments of the Nutri-Score NPS proposed in S2 (discounting SFA from nuts) and S3 (discounting energy from nuts) are in synergy with dietary guidelines. They would help to guide consumers towards healthier dietary choices (plain nuts or relevant products with a meaningful and varied nut contents), incentivise food industries to develop products which contain a variety of nuts, and encourage the consumption of products with an improved nutrient profile. Similar to national dietary guidelines, the straightforward modifications to the NPS of the Nutri-Score that we propose will allow all plain nuts to have the best Nutri-Score letter, without any discrimination between the different types of nuts. This would support the consumption of a variety of nuts. Furthermore, it would incentivise the reformulation and future innovations of nut-containing products.

Both S2 and S3 scenarios would help to align Nutri-Score of nut-containing products more fairly, without unduly favouring products with overall low nutritional quality whilst respecting the fundamentals of the NutriScore. Scenario S3 (discounting energy from nuts) may be preferred, because of a higher percentage of products obtaining a ScN corresponding to a better nutritional quality, which enabled some, but not all nut products to shift to an improved Nutri-Score letter. In addition, S3 is the scenario for which the variation in the ScN is the most correlated to the proportion of nuts in the product, both for products with high and low nut content.

Relevant to the stated objective of FOPNL to incentivise reformulation, adjustments that consider nuts more fairly within the system would position some products such that they could deliver realistically achievable reformulation. This could result in a revised letter, which might be considered as a reduction of one to two points of the ScN of the Nutri-Score. A reduction of one point is not insignificant from a nutritional perspective (e.g., 4.5 g of sugars per 100 g). Moreover, products that are far from the boundary driven by inherent nut nutrient content cannot technically be reformulated to also improve their Nutri-Score letter. By adjusting the algorithm to more fairly reflect the nutritional value of nuts, enabling opportunities for some products to be achievably reformulated can be created, in line with the objective of FOPNL, and aligned with food-based dietary guidelines.

Our work has limitations. One limitation is the number of foods products included, which only addressed 68 nuts and nut-containing products. However, specific attention was paid to include food categories having nut-containing products in the European market in 2021, in order to illustrate the variability of these products. The absence of statistical comparisons between scenarios may also be seen as a weakness, but the descriptive data are sufficiently informative for our purpose. Another limitation is that this work remains theoretical and based on modelling only. A consumer study that would evaluate the healthiness perception and purchase intention of nuts and nut-containing products by European consumers is warranted to validate the likelihood that the proposed modification of the NPS of the Nutri-Score can reduce the current gap between recommendations and actual nut consumption.

There are also strengths to our work. One major strength was that the fundamentals of the NPS of the Nutri-Score have been kept in the four tested scenarios. Indeed, not counting energy or SFA from nuts is easy to implement in practice, as the nutritional values for nuts are publicly available on many validated nutrition composition tables. Furthermore, we have ascertained that both adjustments of the NPS of the Nutri-Score will not impact on food products that do not contain nuts.

## 5. Conclusions

There is robust evidence to support the daily consumption of a handful of nuts in helping to reduce chronic disease risk as part of a balanced diet within a healthy lifestyle. Various guidelines promote nut consumption, yet populations in Europe do not achieve the recommended daily handful of nuts. The more we can encourage nuts in a variety of foods, the more nut consumption is likely to increase across people with different dietary patterns. Of note, in the current transition towards a more plant-based diet, whilst plain nuts remain the reference, nut-containing products can support intakes of plant protein.

Penalising nuts and nut-containing products due to their SFA and energy contribution, with a potential consequent adverse effect on the Nutri-Score letter, does not encourage the purchase or consumption of these nutrient-dense foods. Further, a Nutri-Score letter of “B” or “C” label on plain nuts could spark consumer confusion over the healthiness of nuts within a balanced eating pattern. Healthcare professionals have a key role to play in advising the public to eat a range of nutritious foods, including nuts. A Nutri-Score that appropriately accommodates the positives aspects of nuts, as shown by scientific evidence and recognised in food-based dietary guidelines, and that reflects the nut content of food products, could help professionals impart clearer and non-conflicting advice on how to include nuts within an overall healthy diet and lifestyle.

To conclude, adjustments of the NPS of the Nutri-Score, such as discounting the SFA or energy density from nuts, could be an efficient and easy-to-implement way to better inform the consumer about the healthiness of plain nuts and nut-containing products and incentivise development of healthier nut-containing products. Finally, these slight adjustments to the Nutri-Score result in slightly improved scoring, which contribute to better informing consumers on the nutritional value of nut-containing products, and which might ultimately help to reduce the gap between nut recommendations and actual consumption in Europe.

## Figures and Tables

**Table 2:** Number of products and mean nuts content, nutritional score of the Nutri-Score NPS and Nutri-Score letter included in the nuts and nut-containing products database by food category.

Food Category	Number of Products	% of Nuts (Mean ± SD)	ScN (Mean ± SD)	Nutri-Score Letter Range
Cereal bars	16	31.2 ± 18.6	11.7 ± 5.0	B–D
Breakfast cereals	7	9.9 ± 4.1	2.3 ± 4.5	A–C
Cakes and pastries	2	13.1 ± 2.7	23.0 ± 0.0	E
Sweet biscuits	3	9.2 ± 4.1	19.0 ± 8.7	C–E
Chocolate and chocolate-based products	6	28.9 ± 26.1	20.0 ± 4.3	D–E
Non-chocolate confectioneries	2	29.5 ± 0.7	14.5 ± 2.1	D
Ice creams	4	13.8 ± 5.9	17.2 ± 1.3	D–E
Crackers	2	34.0 ± 5.6	15.5 ± 5.0	D–E
Plant-based alternatives to dairy	2	41.8 ± 38.6	5.5 ± 9.2	A–D
Cheese and related products	1	5	13	D
Plain nuts	9	100 ± 0.0	−0.22 ± 5.0	A–C
Salted nuts	7	96.4 ± 1.5	4.0 ± 5.0	A–D
Spreads (nut “butters”)	2	100 ± 0.0	−1.5 ± 3.5	A–B
Coated nuts	3	62.0 ± 2.6	12.0 ± 2.0	C–D
Nut mix	2	100 ± 0.0	0.0 ± 2.8	A–B
	68	47.2 ± 37.7	9.4 ± 8.4	A–E

**Table 3:** Variation in the nutritional score of the Nutri-Score (ScN) and modification of the Nutri-Score letter following the application of S1, S2, S3 and S4 on the sample of 68 nuts and nut-containing food products.

	S1	S2	S3	S4
ScN variation (points) Mean ± SD (range)	−0.4 ± 1.4 (−8–0)	−3.3 ± 3.1 (−10–0)	−3.8 ± 3.0 (−9–0)	−3.4 ± 2.3 (−9–0)
Number (and percentage) of products that obtained an ScN corresponding to a better nutritional quality by a decrease of at least 1 point	11 (16%)	52 (76%)	62 (91%)	60 (88%)
Variation in ScN of products that obtained an ScN corresponding to a better nutritional quality (points) Mean ± SD	−2.3 ± 2.8	−4.3 ± 2.9	−4.1 ± 2.9	−3.9 ± 2.0
Number (and percentage) of products that obtained a better Nutri-Score letter	3 (4%)	25 (37%)	27 (40%)	31 (46%)

**Table 4:** Linear regression between nuts’ content as a percentage of weight of 68 selected nuts and nut-containing products and the nutritional score of the Nutri-Score with the initial nutrient profiling system and following the application of four modification scenarios (S1, S2, S3, S4).

	Initial	S1	S2	S3	S4
Correlation between nut content and variation in the ScN (R^2^)	/	0.01	0.67	0.86	0.41
Regression coefficient between nut content and variation in the ScN	/	−0.0038	−0.068	−0.074	−0.040
Correlation between nut content and ScN (R^2^)	0.34	0.36	0.54	0.55	0.52

**Table 5:** Correlations between nut content and nutritional score (ScN) and its variation (change in ScN) following the application of four modification scenarios (S1, S2, S3, S4). Correlations were calculated for products with a nut content below 40% in weight (36 products) or above 40% (32 products).

	S1		S2		S3		S4	
	Less than 40% Nuts	More than 40% Nuts	Less than 40% Nuts	More than 40% Nuts	Less than 40% Nuts	More than 40% Nuts	Less than 40% Nuts	More than 40% Nuts
R^2^ between nut content and variation in the ScN	0.51	0.60	0.23	0.20	0.46	0.68	0.023	0.20
R^2^ between nut content and ScN	0.0043	0.026	0.00036	0.73	0.00095	0.72	0.0043	0.76

## Data Availability

ScN and all the variables needed for calculations the ScN of the Nutri-Score for each food product included in this study are available in Supplementary Materials (Table S3). The methods part of the manuscript provides all details for the calculations performed for each scenario.

## Supplementary Materials

The following supporting information can be downloaded at: https://www.mdpi.com/article/10.3390/nu14132668/s1, Figure S1: Methodology for the calculation of the nutritional score of the Nutri-Score and attribution of the corresponding letter for solid foods; Table S1: Nuts in European national dietary guidelines; Table S2: Nuts in dietary guidelines from scientific or healthcare societies, example of the United Kingdom. Table S3: Database containing the nutritional composition of all products used in this study together with calculation of the ScN with the initial Nutri-Score algorithm and with the scenarios S1 to S4.
