# Supplementary material for: A Slight Adjustment of the Nutri-Score Nutrient Profiling System Could Help to Better Reflect the European Dietary Guidelines Regarding Nuts
Source: Nutrients. 2022 Jun 27;14(13):2668. doi: 10.3390/nu14132668 (PMC9268614; doi:10.3390/nu14132668)
Supplement: Supplementary file 1 [file nutrients-14-02668-s001.zip › Supplemental Figure S1.pdf]

Negative compounds (N)

Points	Energy density (kJ/100g)	Sugars (g/100g)	Saturated * fatty acids (g/100g)	Sodium (mg/100g)[1]
0	≤ 335	≤ 4.5	≤ 1	≤ 90
1	> 335	> 4.5	> 1	> 90
2	> 670	> 9	> 2	> 180
3	> 1005	> 13.5	> 3	> 270
4	> 1340	> 18	> 4	> 360
5	> 1675	> 22.5	> 5	> 450
6	> 2010	> 27	> 6	> 540
7	> 2345	> 31	> 7	> 630
8	> 2680	> 36	> 8	> 720
9	> 3015	> 40	> 9	> 810
10	> 3350	> 45	> 10	> 900

Positive compounds (P)

Points	Fruits, vegetables, pulses, nuts, and rapeseed, walnut and olive oils (%)	Fibre (g/100g)	Protein (g/100g)
0	≤ 40	≤ 0.9	≤ 1.6
1	> 40	> 0.9	> 1.6
2	> 60	> 1.9	> 3.2
3	-	> 2.8	> 4.8
4	-	> 3.7	> 6.4
5	> 80	> 4.7	> 8.0

*For added fats, SFA column is replaced by the ratio SFA/lipids below

Points	Ratio total saturated fatty acids/lipids (%)
0	<10
1	<16
2	<22
3	<28
4	<34
5	<40
6	<46
7	<52
8	<58
9	<64
10	≥64

Nutritional score (ScN) = N – P *

*Except if Sum (N) ≥11 pts & Fruit & veg < 5 → **ScN = N – Sum (Fruit & veg + Fibres)**

ScN (points)	Letter	Colour
Min to -1	A	Dark green
0-2	B	Light green
3-10	C	Yellow
11-18	D	Light orange
19 to Max	E	Dark orange

Grid to attribution Nutri-Score Letter depending on ScN points for solid foods

Figure S1. Methodology for the calculation of the nutritional score of the Nutri-Score and attribution of the corresponding letter for solid foods.
